# Sustainable production of silver chloride nanoparticles from desert flora for biomedical applications with multifunctional biological activities

**DOI:** 10.1038/s41598-025-20170-y

**Published:** 2025-09-23

**Authors:** Bothena M.A. Eltayeb, Ezzat H. Elshazly, Hesham A. Aboelmagd, Sabry A. H. Zidan, Abdel Kareem S. H. Mohamed

**Affiliations:** 1 Department of Microbiology and Chemistry, Faculty of Science, Assiut University, Assiut, 71524 Egypt; 2 Department of Botany and Microbiology, Faculty of Science, Assiut Branch, Al-Azhar University, Assiut, 71524 Egypt; 3 Department of Pharmacognosy, Faculty of Pharmacy, Assiut-Branch, Al-Azhar University, Assiut, 71524 Egypt

**Keywords:** AgCl-NPs, HSV-1, Euphorbia sanctae-catharinae, MTT assay, Cytotoxicity, Antimicrobial, Gram-positive, Gram-negative bacteria, Biochemistry, Biological techniques, Biotechnology, Microbiology

## Abstract

In this work, *Euphorbia sanctae-catharinae* leaf extract is used for the first time to biosynthesize silver chloride nanoparticles (AgCl-NPs) via a green synthesis approach. The formation and properties of the AgCl-NPs were confirmed using various characterization techniques: UV–Vis spectroscopy showed a characteristic absorption peak at 430 nm, indicating nanoparticle formation; X-ray diffraction (XRD) analysis revealed a crystallite size of approximately 24 nm; transmission electron microscopy (TEM) showed predominantly spherical nanoparticles with sizes ranging from 20 to 50 nm; and Fourier-transform infrared spectroscopy (FTIR) identified functional groups from the plant extract involved in nanoparticle stabilization. The MIC values were 62.5 µg/mL for (*S. aureus*, *E. coli*, *E. faecalis*,* S. faecalis*,* E. aerogenes)*, 250 µg/mL for (*S. epidermidis*,* K. pneumoniae*,* R. ornithinolytica)*,* and* 125 µg/mL for *P. aeruginosa. The* MBC values were 500 µg/mL for (*R. ornithinolytica*,* S. faecalis*,* K. pneumoniae*,* S. epidermidis)*,* and* 250 µg/mL for *(S. aureus*,* E. coli*,* E. faecalis)*,* and* 125 µg/mL for *E. aerogenes.* Tetracycline’s synergistic actions increased antibacterial effectiveness by 21.4–47%. Furthermore, at a non-toxic dose (MNTC: 31.25 µg/mL), AgCl-NPs showed strong antiviral activity against HSV-1, preventing viral multiplication by 74%. These results demonstrate the potential of AgCl-NPs produced from *Euphorbia sanctae-catharinae* as a sustainable substitute for fighting viral infections and antibiotic resistance.

## Introduction

These days, metal and metal oxide nanoparticles’ distinct biological characteristics have demonstrated great promise in the treatment of a wide range of diseases^1,2^. such as antioxidant, antiviral, antifungal, anticancer, and anti-inflammatory properties—mainly due to their high surface-to-volume ratio, improved cellular absorption, and capacity to produce reactive oxygen species (ROS). Ag–ZnO and Fe₂O_2_@CuO@ZnO nanocomposites, for instance, have shown strong antibacterial and anticancer properties in vitro, with MIC values as low as 7.8 µg/mL and IC₅₀≈53 µg/mL, respectively. Additionally, in models of cellular aging, metal oxides such as CeO₂ NPs have demonstrated ROS scavenging antioxidant activity, which helps to protect against oxidative stress^3^. One of the transitional elements, silver nanoparticles (AgCl-NPs), are a special kind of nanometal particle that finds widespread application in medical science and engineering^4^. AgCl-NPs have demonstrated important therapeutic properties, including wide-ranging antimicrobial, anticancer, anti-inflammatory, antioxidative, and diabetic medications action^5,6^. The green synthesis of AgNPs has attracted a lot of attention due to their environmental friendliness and potential for further processing for use in industry^7,8^. Because it eliminates the need for laborious and specialized procedures including isolation, culture management, and many purification stages, the generation of NPs from plant extracts is superior to intracellular synthesis using microorganisms. Due to these considerations, researchers have mostly concentrated on developing environmentally friendly techniques that make use of a range of plant parts, such as leaves^9–14^, peels^15,16^, flowers^14^, fruits^16^, and roots^15^. Proteins, polyphenols, flavonoids, ascorbic acid, and terpenoids are among the many compounds included in plant extracts that are essential for the absorption of metal ions, the reduction of precursor salts, and the natural antibacterial properties of capping agents^17–20^. Plant extracts are an effective and safe alternative for nanoparticle synthesis, offering an environmentally friendly method free from toxic chemicals. This approach is cost-effective and faster than microbial methods, as plants can reduce and stabilize nanoparticles using their diverse bioactive compounds. Additionally, it does not require high temperatures or pressures, making it ideal for sustainable industrial applications^21,22^.

One of the most intriguing and potential sources of medicinal plants is the Sinai Peninsula, and Saint Katherine in particular. Many scientists, particularly biologists and physiochemists, are interested in conducting more research on the therapeutic plants found in Sinai since they have demonstrated distinctive phytochemicals with strong biological activity. It is commonly recognized that the chemical and biological variety of Euphorbia species varies throughout the world. The primary components of plants in this genus are diterpenes, which include myrsinols, tiglianes, ingenanes, lathyranes, jatrophanes, premyrsinanes, and abietanes. The various extracts and isolated metabolites of these plants have been shown to have a variety of bioactivities, including cytotoxic, antiviral, and antibacterial properties^23^.

Antimicrobial resistance (AMR) is a major issue impacting public health and global development. According to estimates, bacterial AMR directly caused 1.27 million fatalities and contributed to 4.95 million deaths globally in 2019^24^. By 2050, antibiotic resistance is expected to kill more people than cancer, which is terrible^25^. When bacteria and fungi become resistant to the drugs meant to destroy them, this is known as antimicrobial resistance. One of the main causes of this resistance is the overuse of antibiotics, which has allowed bacteria to create biofilms that are solidified in the extracellular matrix and prevent drugs from entering cells^26,27^. Treatments using ultrasound (US) and slightly acidic electrolyzed water (SAEW) were successful in eliminating Listeria monocytogenes biofilms. Additionally, even mild exposure to antibiotics can change microbiota and cause antibiotic resistance^26^. Because drug-resistant bacteria are a global concern to human, animals, and environmental health, there is an urgent need for novel and powerful biofilm components. Silver nanoparticles (AgNPs) may one day be created as a new class of antimicrobial agent to treat bacterial infections, particularly those that are resistant to numerous medications, due to their strong antibacterial activity^28^. Silver nanoparticles show synergistic antibacterial activity against Salmonella typhi, *S. aureus*, and *E. coli* when combined with penicillin, amoxicillin, ampicillin, clindamycin, kanamycin, chloramphenicol, erythromycin, and vancomycin^29^. Numerous investigations have demonstrated that Ag-NPs are an effective biocidal agent against a range of Gram-positive and Gram-negative bacteria, including fungal pathogens and bacteria that are resistant to multiple drugs^30,31^.

One of the main causes of illness and mortality in the globe is viruses. Viral infections have a major effect on the global economy because they can lead to hepatitis viruses and other herpes virus disorders including shingles, chickenpox, mononucleosis infection, genital herpes, viral encephalopathy, or herpes keratitis^32^. HSV-1 is one of the most common viruses, affecting between 50% and 90% of people globally. Roughly 90% of people have HSV-1^33^. The best treatment for HSV-1 infection is thought to be acyclovir (ACV)^34^.

According to some study, long-term usage of ACV might result in a number of negative side effects, such as skin rashes, diarrhea, vomiting, nerve poisoning, and stomach pain^35^. There is growing evidence that excessive usage of nucleoside analogs, including ACV, may encourage the development of treatment-resistant HSV-1 mutants^36^. Therefore, the rise of prevalent drug-resistant HSV-1 strains has created a considerable obstacle to efficiently treating HSV-1 infection^37^. Research and the development of innovative and effective anti-HSV-1 medications are urgently needed because the HSV vaccine is not yet ready for the global market and HSV-1 is growing more resistant to conventional treatments. Its ongoing development in recent years has created amazing prospects for the development of antiviral drugs that use nanoparticles. Ag-NPs efficiently suppress a number of human pathogenic viruses, including adenovirus, Herpes Simplex Virus Types I and II (HSV-I and HSV-II), and Hepatitis B Virus (HBV)^38^.

With an emphasis on the inhibitory effect of AgCl-NPs on Gram-positive and Gram-negative bacteria and viral replication for HSV-1 and adenoviruses as a model, this work synthesized safe, therapeutic, and cytotoxic concentrations of AgCl-NPs for in vitro determination using an environmentally friendly nanotechnology method. The utilization of silver chloride nanoparticles as antivirals against adenovirus and HSV-1 makes this work novel due to the rapid rate of virus mutation.

## Materials and methods

### Chemicals and materials

AgNO3 (Sigma-Aldrich, India), de-ionized water (Merck, South Africa), and silver nitrate. Before being utilized, every piece of glassware used in the experiment was properly cleaned, rinsed with deionized water, and dried. To prevent any photochemical reactions, the silver-related process was conducted in the dark.

All investigations, including the creation of medium for bacterial cell growth, used pure and analytical-grade substances.

### Preparation of *Euphorbia sanctae-catharinae* Extract

A sufficient quantity of *Euphorbia Saint Catherine’s* aerial portion was gathered from the Saint Catherine desert in South Sinai, Egypt during the flowering stage in April 2022 under the permission of the Al-assiuty protectorate for the purposes of scientific research. The collection of the plant *Euphorbia sanctae-catharinae* comply with the IUCN Policy.

A Voucher specimen with the code (ESC-2) has been deposited in the herbarium of the Botanical Collection, Pharmacognosy Department, Faculty of Pharmacy at Al-Azhar University, Assiut, Egypt. The identification and authentication of the plant were kindly performed by Prof. Dr. Abdelkareem Sayed Hussein, professor of botany and plant taxonomy at the Faculty of Science Al-Azhar University, Assiut, Egypt.

In the shed, the collected parts were left to air dry until they were totally dry. The dried herbs were ground into a dry powder weighing about 250 g. The air-dried powders were defatted by macerating them with 3 L of n-hexane three times over the course of an overnight period at room temperature. After the solvent was filtered through Whatman filter paper, the extracted material was allowed to dry. Then, at room temperature, the dried marc was macerated in methanol-water (7:3, v/v) until it was eliminated (3 L, each time, overnight). A concentrated dry hydro alcoholic extract (24 g) was obtained by filtering the solvent using Whatman filter paper and then using a rotatory evaporator to evaporate it at 40 °C under reduced pressure. The following components were screened for using known methods in two grams of the hydroalcoholic extract: anthraquinones, flavonoids, tannins, alkaloids and/or nitrogenous bases, sterols and/or triterpenes, carbohydrates and/or glycosides, and saponins. The results show that, except for anthraquinones, the plant is rich in all of the previously indicated components. The remaining 22 g of the hydro alcoholic extract were maintained in a refrigerator till the current investigation was carried out.

### Green synthesis of AgCl-NPs

AgCl-NPs were created using aqueous *Euphorbia sanctae-catharinae* extract and 1 mM AgNO_2_. They were created from seeds by combining four milliliters of aqueous seed extract with 96 milliliters of AgNO_2_ that had been exposed to sunlight. The solution’s color changed continuously as the aqueous seed extract changed from yellow to dark brown^28^.

### Characterization of AgCl-NPs by Ultraviolet-visible (UV-visible) analysis

To illustrate the full biological reduction of AgNO_2_ to AgCl-NPs, one milliliter of the sample suspension was diluted with two milliliters of pure water. A Shimadzu UV sensor 1800 UV-visible spectrophotometer, which has a scan spectrum spanning from 200 to 700 nm, was also used to measure the material’s spectra.

### AgCl-NPs’ characteristics using X-ray diffraction (XRD) analysis

In this investigation, analytical XRD was used using a monochromatic filter in the 2-range from 10 to 80, an operating voltage of 40 kV, and an average scanning time of 20 min1. It has been used to identify phases and describe the structure of nanoparticles.

### Analysis of AgCl -NPs using transmission electron microscopy (TEM)

TEM was used to examine the size and shape of the generated Agcl-NPs. A 200 kV ultra-high-resolution TEM (JEOL, JEM 2100 h with EELS) was used for the experiment. A drop of the nanoparticle suspension was applied to 300 mesh copper grids that had a tiny layer of carbon applied to them. After allowing the grids to air dry, TEM was used to visualize the size and shape of the nanoparticles^28^.

### Characterization of AgCl-NPs by Fourier transform infrared spectroscopy (FTIR) analysis

The FTIR spectra of specimens of the *Euphorbia sanctae-catharinae* extract AgCl-NPs were analyzed. The FTIR analysis used KBr pellets, which had measurements between 400 and 4,000 cm-1. To identify the distinct functional groups contained in the samples, the different vibration modes were identified and assigned.

### Evaluation of AgCl-NPs’ antibacterial activity

Inhibition zone (IZ) measurement using the Agar-well diffusion method. Dimethylsulphoxide (DEMTHO) was used as a negative control in a slightly altered version of the agar-well diffusion test, as detailed by Hsouna et al.^39^. The plates were maintained at + 4 ◦C for two hours to facilitate the diffusion of the compounds being studied in the agar^40^. At 37 °C, the incubation procedure lasted for 24 h. The diameter of the inhibition zone (IZ) that developed around the well was measured in order to assess the bactericidal properties; the mean value was calculated for three duplicate trials.

### Estimation of MIC & MBC by Macro-dilution method

The apparent growth of the bacteria in Mueller Hinton broth (MHB) media was assessed using the standard broth dilution method (Clinical and Laboratory Standards Institute, CLSI M07-A9)^41^. In a liquid growth medium that was split among tubes with a capacity of around 2 mL, AgNPs were first serially diluted twice. The values range from 1000 to 31.25 µg/mL (macro-dilution). A bacterial inoculum set to (108 CFU/mL) was then injected into each tube, and the bacterial suspension was created in the same medium, 0.5 McFarland’s starting point. Only inoculated broth is present in one tube, known as the control tube, which is incubated for twenty-four hours at 37 °C. The minimal inhibitory concentration (MIC) endpoint is the lowest concentration of the tested substances at which little or no growth is observed in the tubes. After subculturing samples from tubes that resulted in negative bacterial growth on the surface of MHA plates for 24 h, the number of viable cells (CFU/mL) can be used to determine the minimum bactericidal concentration (MBC) after broth macro-dilution. The lowest concentration at which 99.9% of the final bacterial inoculation eliminated is known as the bactericidal (MBC) endpoint^42^.

### Evaluation of the effect of a mixture from AgCl-NPs with an antibiotic on the tested bacterial strains

The synergistic antibacterial properties of AgCl-NPs complexes with drugs (tetracycline) were examined using a traditional Agar-well diffusion method. In short, a sterile swab was used to inoculate the surface of MHA plates with a freshly made bacterial suspension that had been adjusted to 108 CFU/mL. A sterile Pasteur pipette was then used to make a hollow (well) of 6 mm in the agar plate. 50 µL of the mixture was then applied to each well in a 1:1 (v: v) ratio, containing 500 µg/mL of AgCl-NPs and 500 µg/mL of tetracycline. The solutions were then sonicated at room temperature for around fifteen minutes.

The synergistic antibacterial effectiveness of the AgNP-antibiotic combinations was assessed using the inhibition zone distances (measured in millimeters) after a 24-hour incubation period at 37 °C^43^.

### The MTT test was used to assess antiviral effects^44^

- All the viruses were in the microbiology section of the Faculty of Medicine for Girls in Cairo, Egypt.
- The Excel software was used for analysis the cytotoxicity and the antiviral effect.

### Assessing the cytotoxicity of AgCl-NPs on VERO cells

Several quantities of the samples under investigation were prepared. The growth medium was extracted from 96-well micro titer plates after the confluent sheet of Vero cells had grown. The cell monolayer was then rinsed twice with rinse media, and the sample under investigation was double diluted in DMEM. Three wells were left as controls, obtaining only preservation medium. The plate was then placed in the incubator at 37 °C and monitored regularly for several days.

An MTT solution was created by Bio Basic Canada Inc. (5 mg/ml in PBS). Each well received a 20 μl injection of the MTT mixture. Place the MTT on a surface that rattles at 150 rpm for five minutes in order to completely mix it throughout the media. To enable digestion, incubate it for four hours at 37 °C with 5% CO2. Discard the media. After wiping the plate on paper towels to remove any remaining residue, suspend it formazan (MTT metabolic output) using 200 μl of DMSO if necessary to thoroughly mix the formazan with the solvent, it should be set up on a shaking table at 150 rpm for five minutes. The optical density is then measured at 560 nm, and the background is then reduced at 620 nm. For each extract, a maximum non-toxic concentration [MNTC] has been determined and used in subsequent biological studies; cell number and optical density ought to be closely related.

### MTT assay protocol

Using 200 μl of media, place 10,000 cells in each of the 96 wells on a tray. Three wells were left empty for the blank checks, and they were incubated overnight at 37 °C with 5% CO2 to allow the cells to attach to the wells. The tested sample and the equal volume (1:1 v/v) of the viral solution should be incubated for 60 min. Pour in 100 µl of the viral/sample solution. Place on a shaking plate, rotate at 150 rpm for 5 min, and then incubate for 1 day at 37 °C with 5% CO2 to enable the virus to start working. Incubate for 1 to 5 h at 37 °C with 5% CO2 after preparing at least 2 ml of MTT solution per 96-well plate at a concentration of 5 mg/ml in PBS. to enable the MTT to be metabolized. Discard the media. To remove any debris, you can use tissue paper to dry the dish if necessary. After re-forming the MTT metabolic product formazan in 200 μl of DMSO, place the Formazan on a shaking surface at 150 rpm for 5 min to fully incorporate it into the solution. After that, read the optical density at 560 nm and lower the background at 620 nm. There must be a direct correlation between cell count and optical density.

### Statistical analysis

The statistical analysis of data comes from the mean and standard deviation (±) of five replicate samples in each experimental group according to statistical validation guidelines. To determine the significance of the difference between the means, a test was performed only within the two significance levels (0.05 and 0.1) for cytological observation.

## Results and discussion

### UV–Visible spectroscopy

**Fig. 1:**
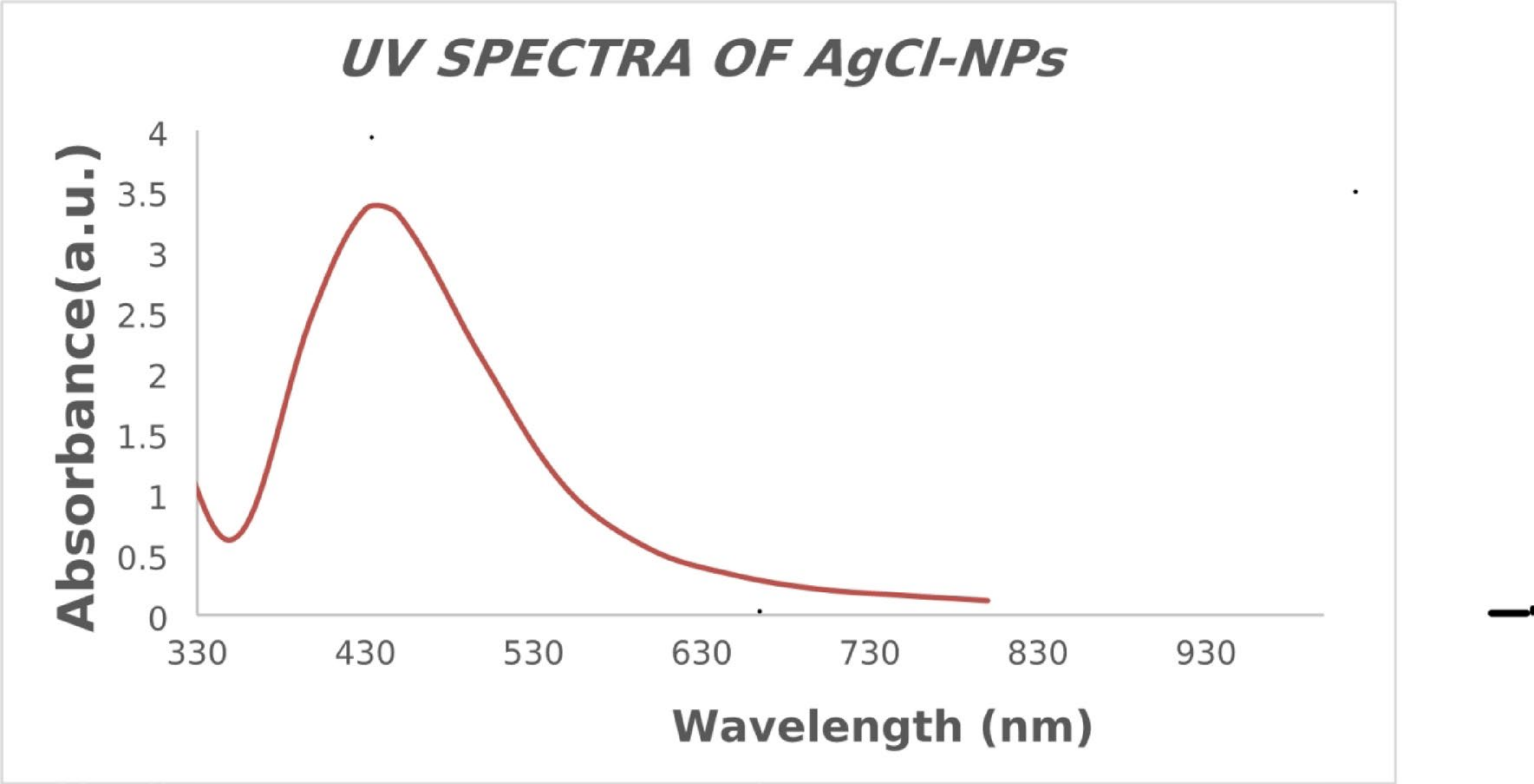
UV-Vis spectrum of biosynthesized AgCl-NPs.

The ensuing reaction mixtures’ UV-Vis spectra demonstrated a progressive increase in the intensity of the localized surface plasmon resonance (LSPR) absorbance region over time. Reports indicate that the production of spherical AgCl nanoparticles (AgCl-NPs) at approximately 430 nm (Fig. 1) was associated with the highest absorbance^47,48^. Moreover, these spectra were consistent with those obtained upon the completion of the reaction, indicating that the reaction had reached completion. This observation suggests that the formation of AgCl-NPs was both efficient and effective, as evidenced by the stable and reproducible absorbance peaks.

**Fig. 2:**
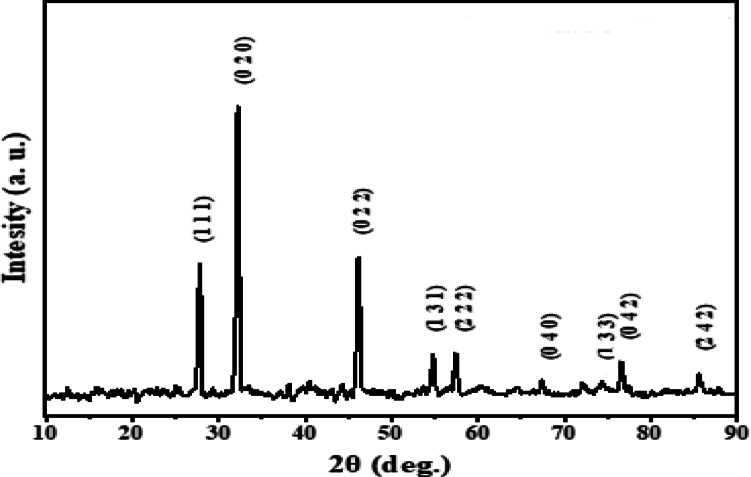
XRD of silver nanoparticles from *Euphorbia sanctae-catharinae* extract the calculated average particle size of *AgCl-NPs* sample is 24 nm.

As shown in (Fig. 2) the diffraction peaks 1 1 1 4 0, 1 3 1, 2 2 2, 0 4 0, 1 3 3, 0 4 2, 2 4 2 are clearly visible in Fig. 1. These peaks correspond to 2θ at 25.50, 27.87, 32.25, 38.11, 46.23, 54.85, 57.49, 67.4214, and 76.61, in that order. The pattern’s high and thin diffraction peaks are caused by the AgCl nanoparticles’ exceptional crystallinity. There are no observable impurity peaks that correspond to the value given by JCP No. 96-901-1667. The average crystallite size (D) has been calculated using Scherrer’s formula^45^:

1 $$\text{D}=\frac{\text{k}\:{\uplambda\:}}{{{\upbeta\:}}_{\text{h}\text{k}\text{l}}\text{C}\text{o}\text{s}{\uptheta}}$$

Bragg’s refractive angle, the wavelength of the used X-ray beam, the whole width at half of the highest intensity of the diffraction peak in (radian), and the variables k, βhkl, λ, and θ in this instance, and shape factor = 0.9. The crystallite is about 24 nm in size.

### FTIR analysis

**Fig. 3:**
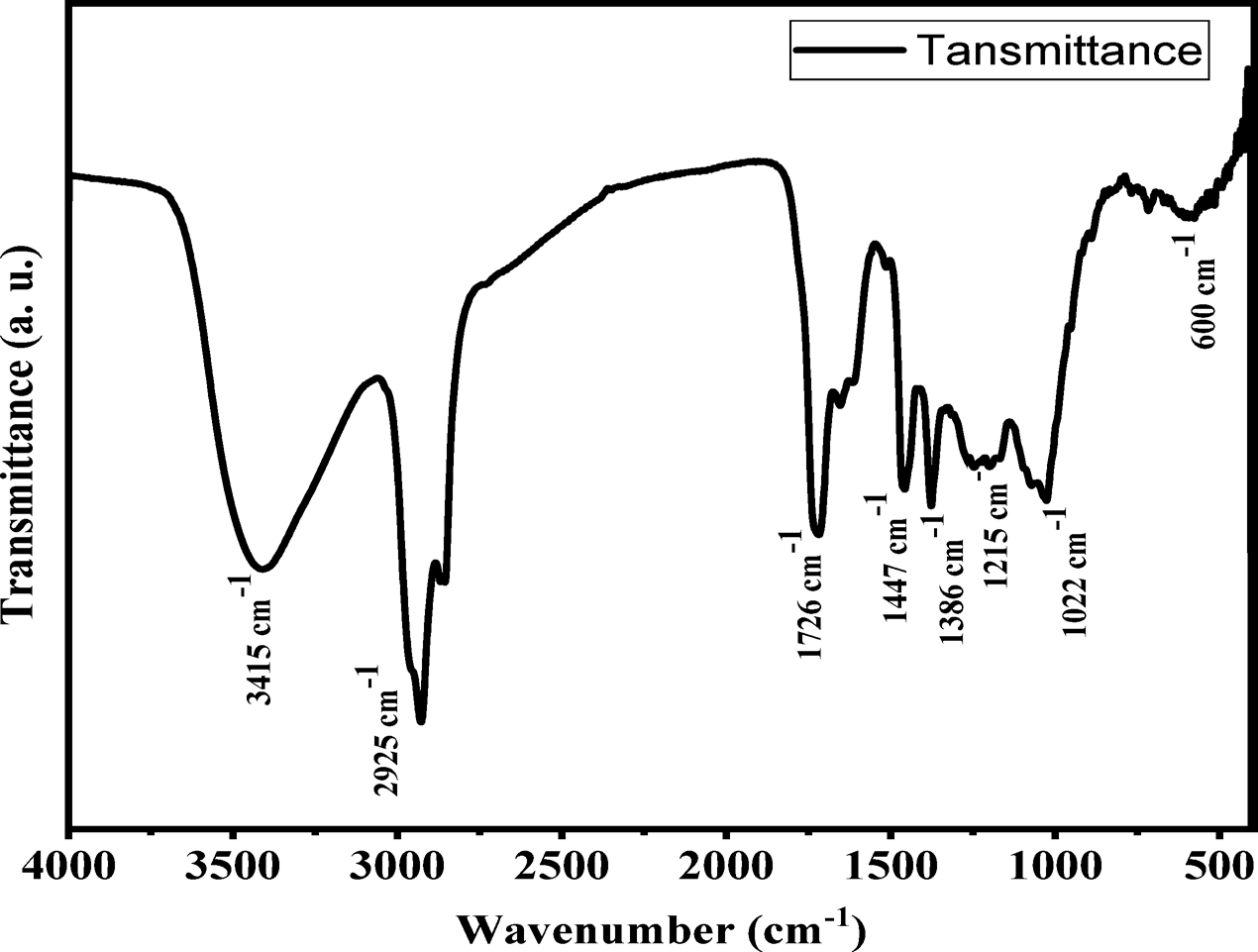
FTIR analysis AgCl-NPs from *Euphorbia sanctae-catharinae* extract.

FTIR analysis was used to determine which chemicals in the extract were linked to the biosynthesized nanoparticles (Fig. 3).

Alkyl halides were identified using the C–Cl stretching vibrations seen at 600 cm^−1^^46^. Around 2925 cm^−1^, C–H stretching vibrations were discovered, which are suggestive of aromatic components^47^. The OH, –C=O functional groups found in phenolic components and alcohols with strong hydrogen bonds were identified using the absorption bands between 3415 cm^−1^ and 1447 cm^−1^^47^. *Euphorbia sanctae-catharinae*, which contains flavonoids and phenolic compounds, capped the generated AgCl-NPs, as demonstrated by all of these FTIR absorption peaks. These compounds likely stabilized the structure of the biosynthesized nanoparticles while imparting new properties A natural source of bioactive substances such proteins, flavonoids, and phenolics was the leaf extract of *Euphorbia sanctae-catharinae*, a rare and endemic plant species from South Sinai. During the creation of nanoparticles, these substances served as both stabilizing and reducing agents. The presence of functional groups originating from these phytochemicals on the nanoparticles’ surface was verified by FTIR analysis. These biomolecules’ well-known antibacterial and antioxidant qualities should improve the produced nanoparticles’ potential for use in biomedical applications. However, this investigation did not directly compare AgCl-NPs that were chemically generated without the plant extract. In order to gain a better understanding of the role that plant-derived compounds play in adjusting the behavior of nanoparticles and boosting their bioactivity, we advise that future comparative studies assess the impact of this special extract on the physicochemical and biological characteristics of the nanoparticles.

### TEM analysis

**Fig. 4:**
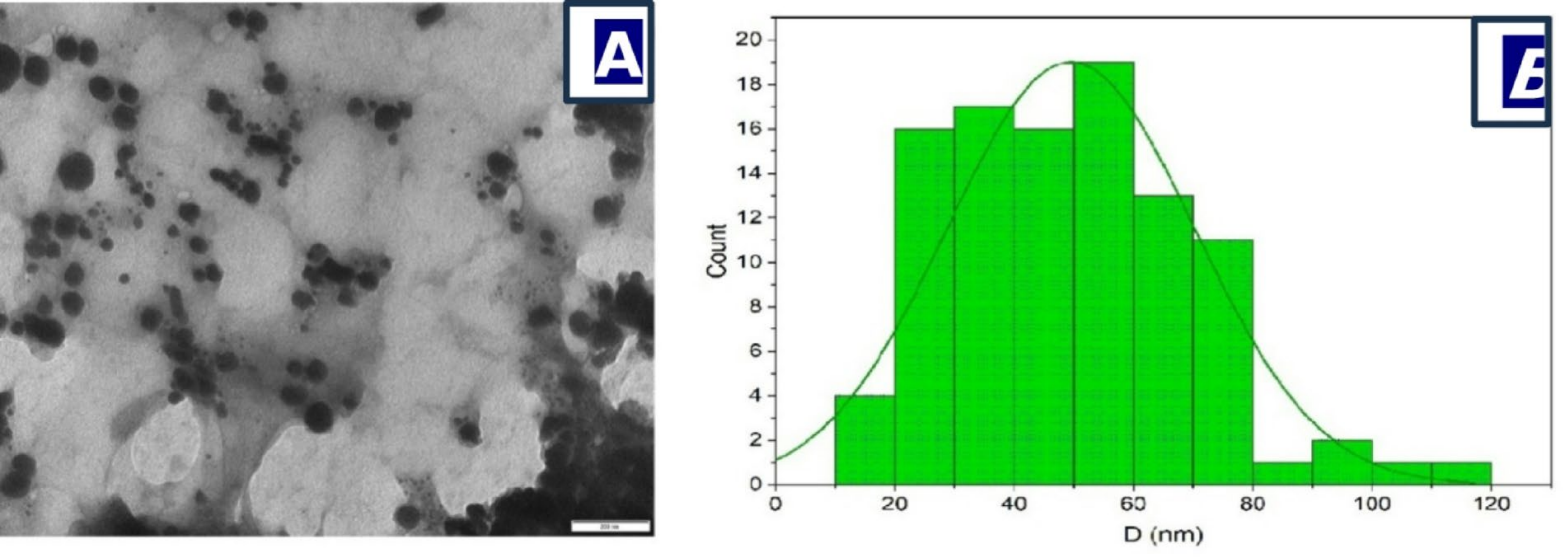
Characterization of the biosynthesized AgCl-NPs: **a** denotes the TEM image and **b** particle size distribution.

The spherical AgCl NPs in Fig. 4a, are uniformly aggregated and, according to a study that examined the crystalline size of the NPs using TEM analysis according to reported elsewhere^48–50^, range in size from 10 to 120 nm on average. The TEM micrograph (Fig. 4a) reveals that the Ag Cl NPs are predominantly spherical to nearly spherical in shape, with some degree of polydispersity. The nanoparticles appear well-dispersed, although mild agglomeration can be observed in certain regions, likely due to high surface energy and Van der Waals interactions. The darker contrast of the particles against the lighter background indicates their crystalline nature and higher electron density compared to the surrounding medium. The scale bar corresponds to 200 nm, indicating that individual particles are generally in the nanometer range.

The histogram curve (Fig. 4b) shows the particle diameter distribution (D, in nm) with a fitted curve indicating a near-normal distribution. The majority of Ag Cl-NPs have diameters between approximately 10 nm and 120 nm, and average size of 49.5 nm (Standard Deviation ± 20.7). The distribution tail extends toward larger sizes up to about 120 nm, suggesting the presence of a small fraction of larger nanoparticles or aggregates. This confirms the TEM observation of slight polydispersity.

### Antibacterial activity

**Fig. 5:**
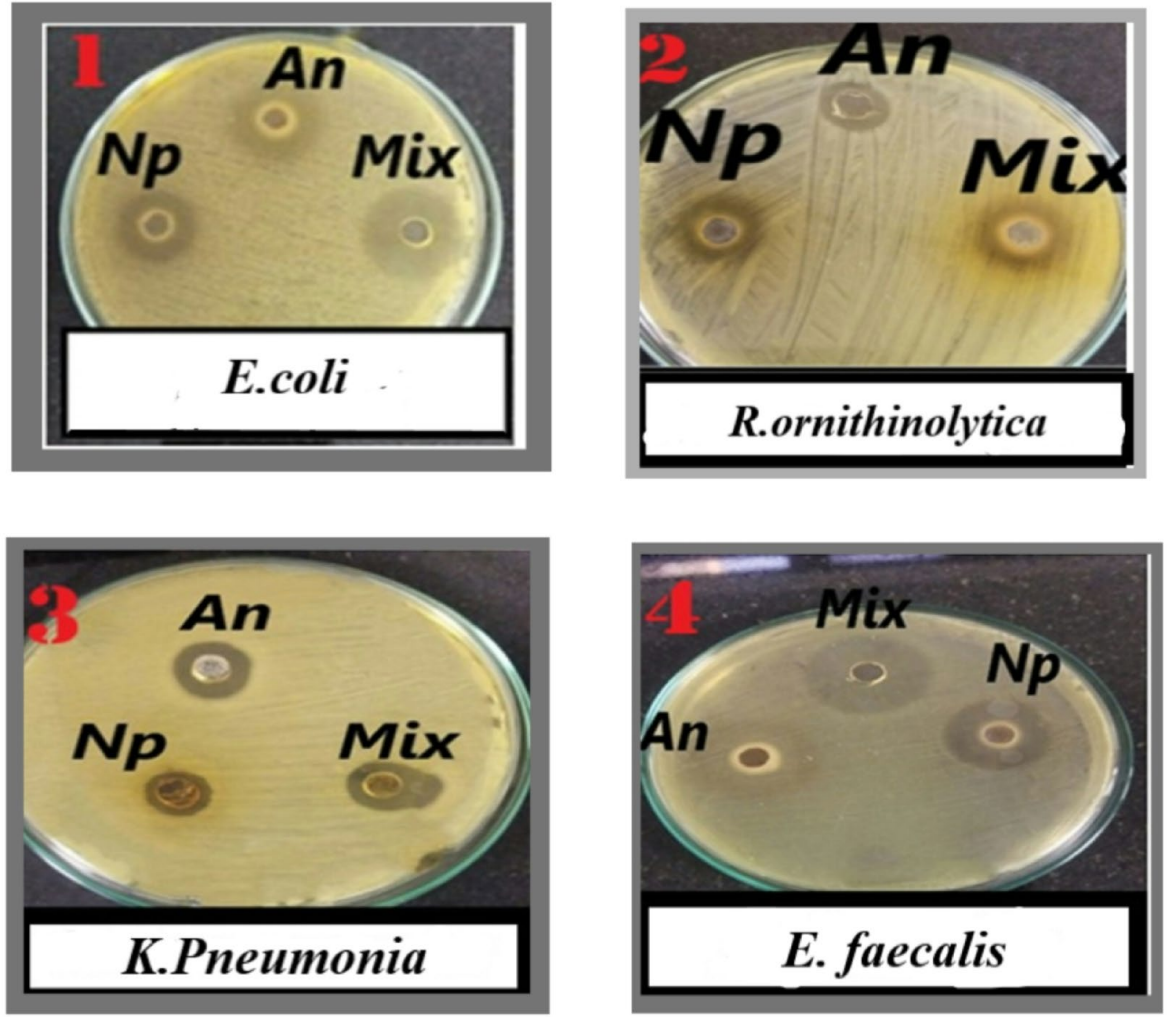
Antimicrobial susceptibility well diffusion method. Zones of inhibition of Mixture of AgCl-NPs and Tetracycline against the selected strains *E. coli* (1), *R. ornithinolytica (*2), *K. Pneumonia* (3), and *E. faecalis* (4).

**Fig. 6:**
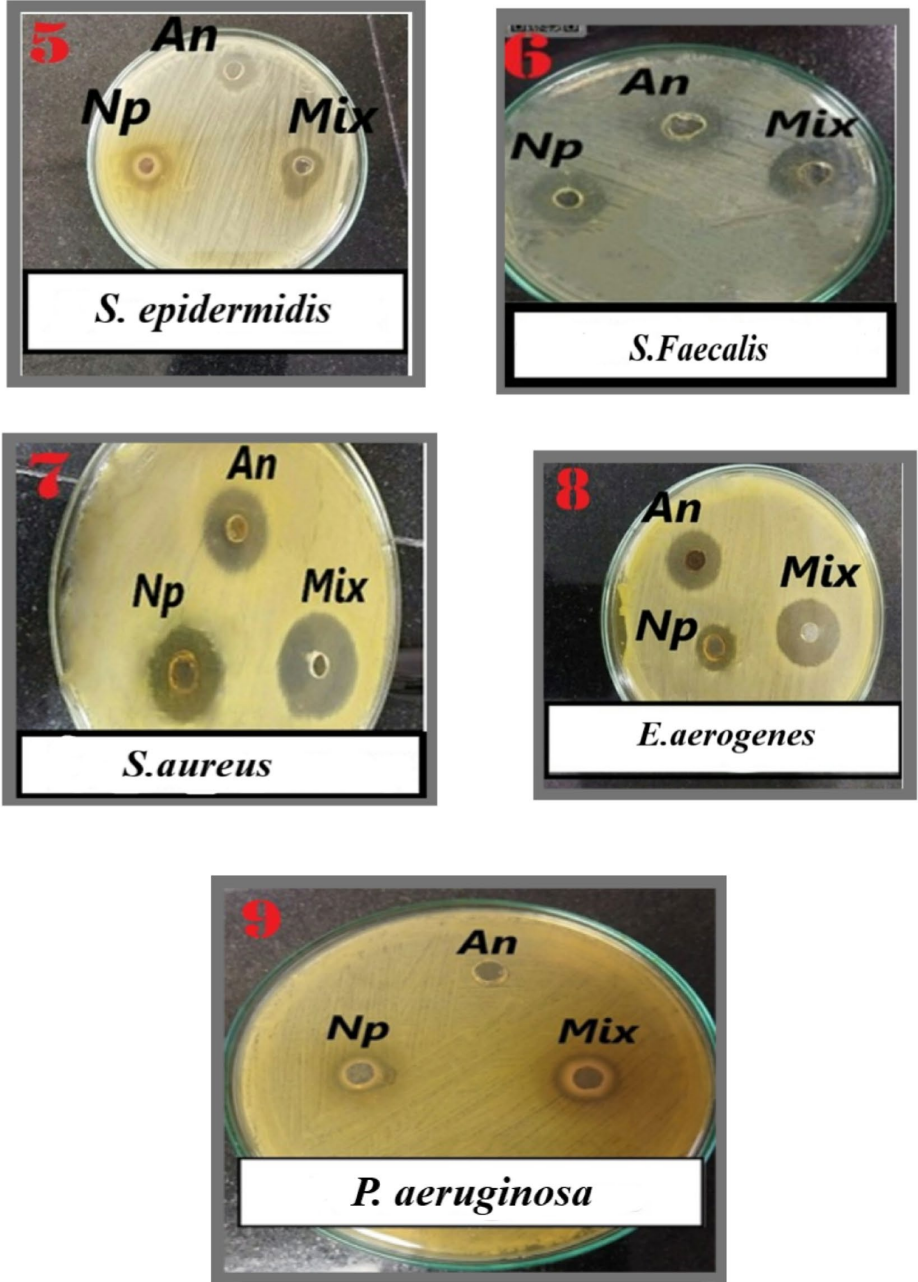
Antimicrobial susceptibility well diffusion method. Zones of inhibition of Mixture of Ag Cl-NPs and Tetracycline against the selected strains *S. epidermidis*(5), *S.Faecalis (*6), *S.aureus* (7), and *E.aerogenes* (8) *P. aeruginosa*. (9).

AgCl-NPs’ antibacterial qualities are well known. In this study, we investigated the synergistic effect of AgCl-NPs combined with tetracycline against the aforementioned bacteria using agar well diffusion, broth microdilution, MIC, and MBC methodologies. The sensitivity of the bacteria was qualitatively evaluated by measuring the inhibition zone (IZ), which is displayed in Figs. 5 and 6, and Table 1. According to our outcomes, AgCl-NPs showed antibacterial activity against every strain that was tested (Table 2). AgCl-NPs have the ability to enter cells and produce reactive oxygen species (ROS) and free radicals^51,52^. The destruction of granularity leads to the suppression of cell growth^53^. However, as Table 3 shows, one of the findings of our investigation was that AgCl-NPs exhibited potent antibacterial activity against *P. aeruginosa*, Tetracycline, on the other hand, had no effect on the bacteria. The three types of *P. aeruginosa* defense mechanisms against antibiotic attack include acquired, adaptive, and intrinsic resistance. *P. aeruginosa’s* innate resistance includes low outer membrane permeability, the creation of enzymes that make antibiotics inert, and the formation of efflux pumps that push drugs out of the cell. Additionally, *P. aeruginosa* can acquire resistance through horizontal transfer of resistance genes or mutational changes^54,55^. With an MBC value of 500 µg/mL and a MIC value of 125 µg/mL, AgCl-NPs demonstrated antibacterial effectiveness against *P. aeruginosa* in our investigation. Carbapenem-resistant *P. aeruginosa* was recently named by the World Health Organization (WHO) as one of three bacterial species where the creation of new antibiotics to treat infections is urgently needed^56^. According to several investigations, such as those by Elnosary et al.^57^, Quinteros et al.^58^, and Khalil et al.^59^, *P. aeruginosa* has shown resistance to AgNPs. It is possible that AgNPs’ effectiveness against P. *aeruginosa* stems from their inability to eliminate excessive ROS and the imbalance between oxidation and antioxidation activities^60^. Other strains showed MBC concentrations ranging from 125 to 500 µg/mL and MIC values ranging from 62.5 to 250 µg/mL, as shown in Table 2. Several studies have shown that uncoated AgNPs often display selective rather than broad-spectrum antibacterial activity. Previous studies have consistently highlighted the limitations of uncoated AgNPs, including their tendency to aggregate, poor stability, and inconsistent biological effects^61,62^. Our Phyto capped AgNPs exhibited MIC values of 62.5 µg/mL against *E. coli* and 125 µg/mL against P. aeruginosa. These results represent an improvement of approximately 38–95% and 37.5–90%, respectively, when compared with the reported MIC ranges of 100–≥1250 µg/mL for *E. coli*^63–65^, and 200–1250 µg/mL for P. aeruginosa^63,64^ using uncoated AgNPs. This clearly demonstrates that Phyto coating not only improves the physicochemical stability of AgNPs but also extends their antimicrobial spectrum to strains that are typically resistant to uncoated nanoparticles. Interestingly, when AgNPs and tetracycline were coupled, antibacterial activity rose by from 21.4 to 47%. The synergistic effects of biogenic AgCl-NPs when mixed with specific antibiotics increase their potential to be used as a free therapy for bacterial infections. It has been shown that nanoparticles plus antibiotic mixtures are more effective than antibiotics alone. These combinations may limit the spread of bacterial strains that are resistant to antibiotics while reducing the duration and dosage needed for antibiotic treatment^66,67^.

**Table 1:** Diameter of inhibition zone (mm) of AgCl-NPs against the tested strains.

Bacterial strains		Concentration (µg/mL)				
		1000	500	250	125	62.5
*E. coli*	Inhibition zone (mm)	23 ± 2.65 a	21 ± 2.08 a	20 ± 2.52 a	18 ± 2.08 ab	17 ± 2.08 b
*S. aureus*		19 ± 2.08 a	18 ± 1.53 a	17 ± 2.08 a	11 ± 1.00 b	9 ± 1.00 c
*R. ornithinolytica*		10 ± 0.58 a	9 ± 1.00 a	8 ± 0.29 a	0 ± 0.0 b	0 ± 0.0 b
*K. Pneumonia*		11 ± 1.00 a	10 ± 0.58 a	9 ± 0.29 a	0 ± 0.0 b	0 ± 0.0 b
*E. aerogenes*		15 ± 1.53 a	13 ± 0.58 ab	12 ± 1.00 b	11 ± 1.0 bc	10 ± 1.00 c
*P. aeruginosa*		11 ± 1.00 a	10 ± 1.00 a	9 ± 0.58 ab	8 ± 0.29 b	0 ± 0.0 c
*S. faecalis*		15 ± 1.53 a	14 ± 1.00 a	13 ± 1.00 ab	12 ± 1.00 ab	9 ± 0.29 b
*E. faecalis*		17 ± 2.08 a	16 ± 2.08 a	15 ± 1.04 ab	14 ± 1.53 ab	13 ± 1.00 b
*S. epidermidis*		12 ± 1.00 a	11 ± 1.00 a	8 ± 0.29 ab	0 ± 0.0 c	0 ± 0.0 c

Values in a raw assigned with different letter denote significant difference (*p* < 0.05).

**Table 2:** MIC and MBC (µg/mL) values of AgCl-NPs against the tested strains.

Bacterial strains	MIC (µg/mL)	MBC (µg/mL)
*E. coli*	62.5 ± 11.7 c	250 ± 17.9 b
*S. aureus*	62.5 ± 11.7 c	250 ± 20.5 b
*R. ornithinolytica*	250 ± 20.5 a	500 ± 36.3 a
*K. Pneumonia*	250 ± 17.9 a	500 ± 24.2 a
*E. aerogenes*	62.5 ± 8.92 c	125 ± 9.23 c
*P. aeruginosa*	125 ± 9.23 b	500 ± 24.2 a
*S. faecalis*	62.5 ± 8.92 c	500 ± 36.3 a
*E. faecalis*	62.5 ± 11.7 c	250 ± 17.9 b
*S. epidermidis*	250 ± 17.9 a	500 ± 24.2 a

Values in a column assigned with different letter denote significant difference (*p* < 0.05).

**Table 3:** Determination the effect of mixture from AgCl-NPs with tetracycline against the tested strains.

Bacterial strains	Inhibition zone (mm)		
	Tetracycline	AgCl-NPs	Mixture
*E. coli*	24 ± 2.52 a	21 ± 2.02 a	26 ± 3.51 a
*S. aureus*	21 ± 2.02 b	19 ± 1.38 ab	23 ± 2.08 b
*R. ornithinolytica*	10 ± 0.29 d	11 ± 0.55 cd	14 ± 1.00 e
*K. Pneumonia*	13 ± 0.63 cd	11 ± 0.55 cd	14 ± 1.00 e
*E. aerogenes*	18 ± 1.29 bc	15 ± 1.05 bc	19 ± 1.51 cd
*P. aeruginosa*	0 ± 0.00 e	13 ± 0.61 c	13 ± 0.61 e
*S. faecalis*	18 ± 1.29 bc	15 ± 1.05 bc	20 ± 1.53 c
*E. faecalis*	20 ± 1.53 b	17 ± 1.21 b	22 ± 2.08 bc
*S. epidermidis*	15 ± 1.08 c	12 ± 0.58 c	17 ± 1.24 d

Values in a column assigned with different letter denote significant difference (*p* < 0.05).

### Antiviral activity

**Fig. 7:**
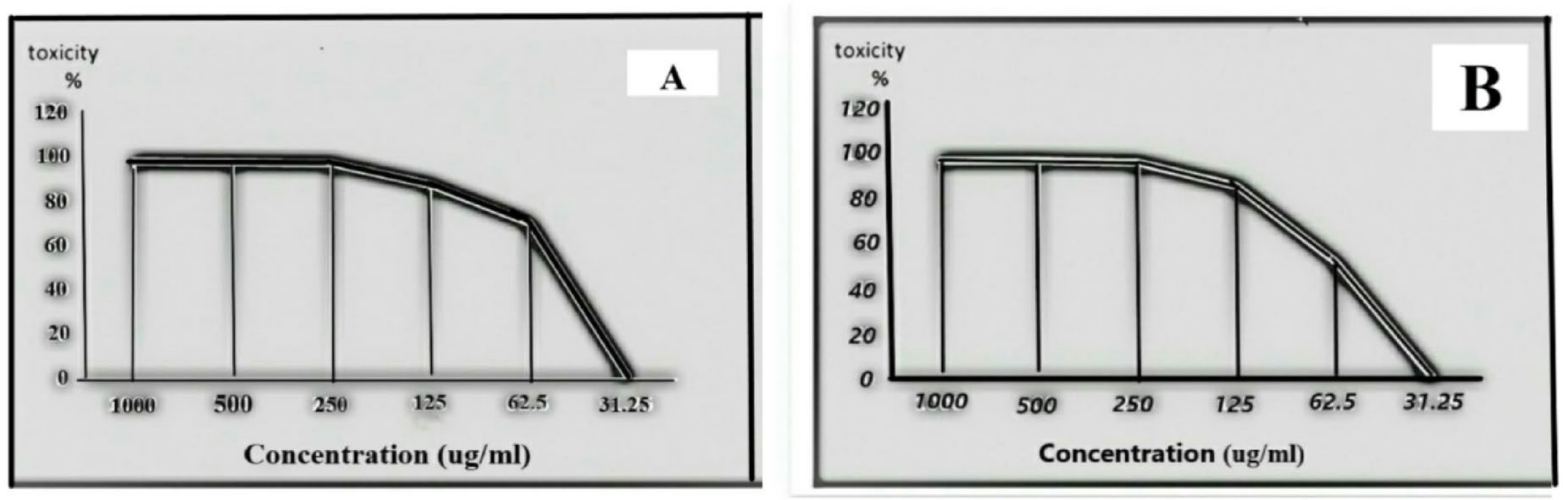
**a** Cell cytotoxicity of plant extract at different concentrations on Vero cell. **b** Cell cytotoxicity of AgCl-NPs at different concentrations on Vero cell.

AgCl-NPs and plant extract were evaluated by generating six concentrations of them in this way: 31.25 ug/mL, 62.5 ug/mL, 125 ug/mL, 250 ug/mL, 500 ug/mL, and 1000 ug/mL, using two-fold dilutions in MEM with FCS. The MTT assay was used to evaluate the cytotoxicity of the nanoparticles in VERO cells in order to verify that the detected doses of AgCl-NPs were safe. The maximum non-toxic concentration [MNTC] of AgCl-NPs and the plant extract is 31.25 ug/ml (Fig. 7).

**Fig. 8:**
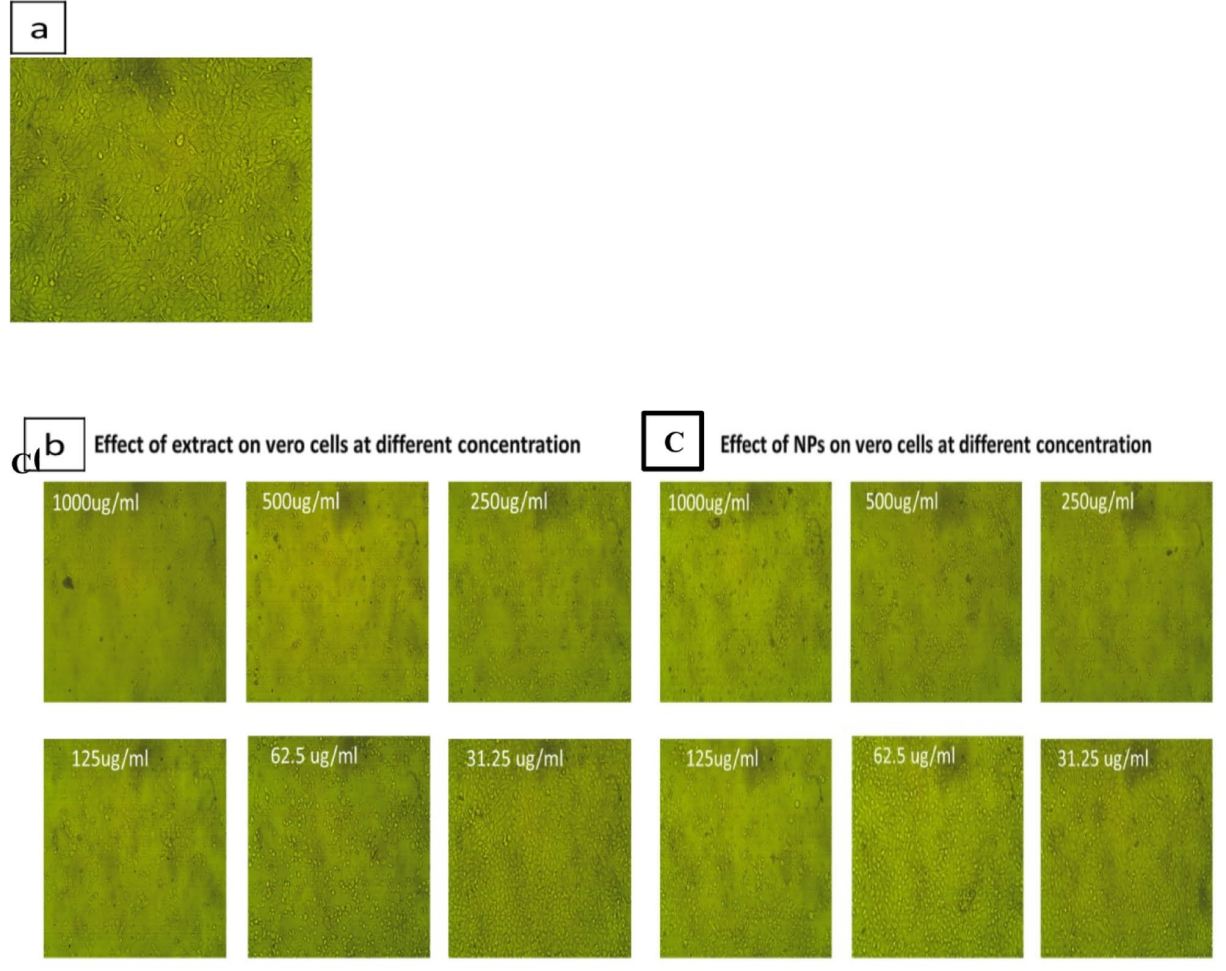
**a** Control Vero cells (x10 magnification). **b** Morphological changes of Vero cells treated with plant extract at different Concentrations (x10 magnification). **c** Morphological changes of Vero cells treated with AgCl-NPs at different Concentrations (x10 magnification).

**Fig. 9:**
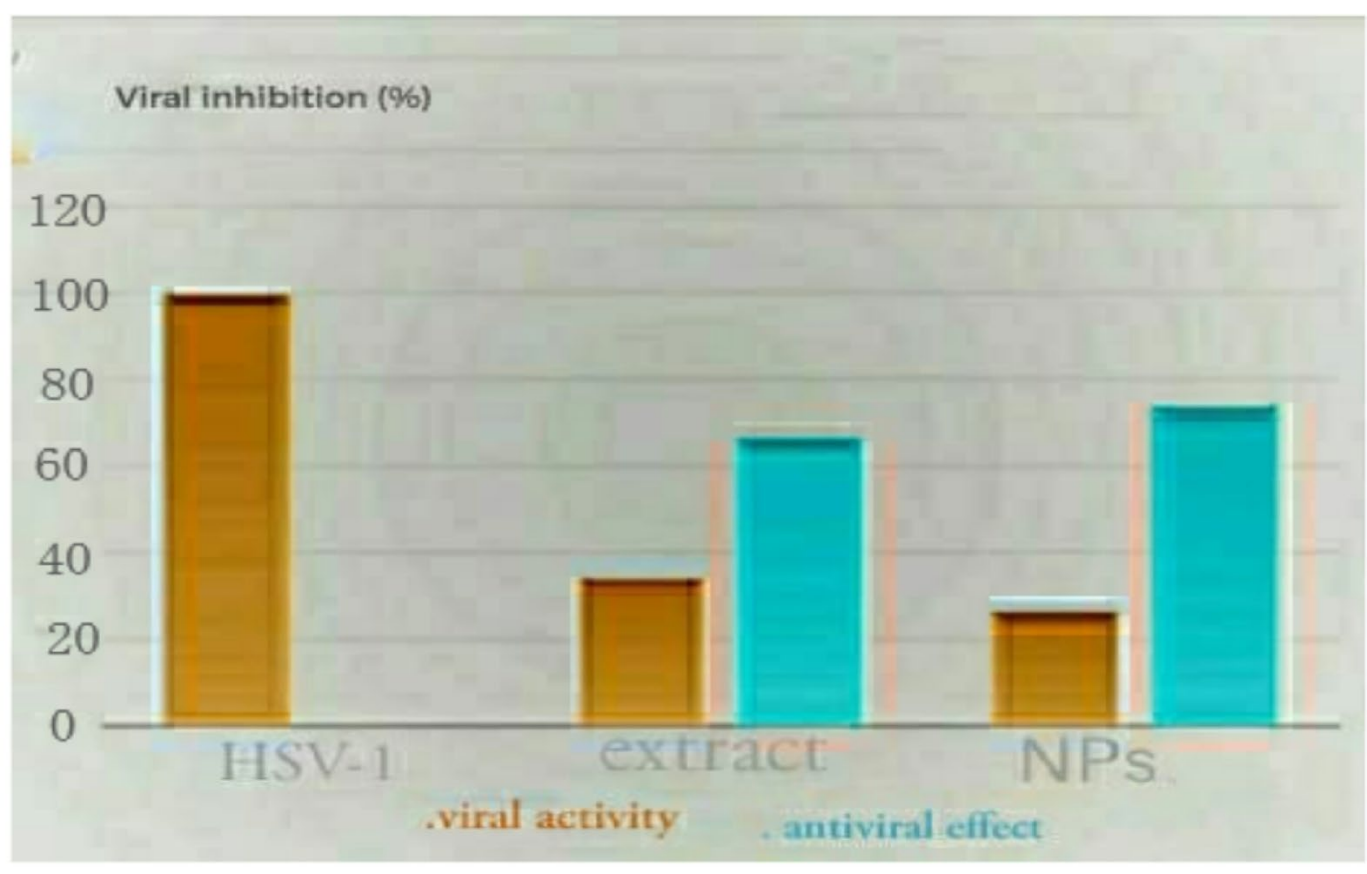
The antiviral activity of free silver nanoparticles (AgCl-NPs) against the herpes simplex virus type 1 (HSV-1) was investigated using the Vero cell line. The antiviral activity of AgCl-NPs was measured as a percentage in comparison to Vero cells infected with plant extract (control) in the MNTC range. Important results have a p-value less than 0.05.

The materials’ cytotoxicity and antiviral qualities against the HSV-1 virus were investigated using the MTT antiviral test methodology (Fig. 8). Studies showed that AgCl-NPs and plant extract were effective against the HSV-1 virus at varying amounts. While the same amount of plant extract modestly inhibited HSV-1, AgCl-NPs at a concentration of 31.25 ug/mL significantly lowered HSV-1 replication with 74%. (Fig. 9). AgCl-NPs demonstrated a cytotoxic concentration (CC 50) of 61.23 ± 0.48 ug/mL, a half maximal inhibitory concentration (IC 50) of 23.03 ± 0.63, and a selective index (SI) of 2.6587. However, Table 4 shows that the plant extract had (SI) of 2.1870, (IC 50) of 24.43 ± 1.02, and (CC 50) of 53.43 ± 0.16.

**Table 4:** Antiviral activity of AgCl-NPs against HSV-1, CC50: cytotoxic concentration, IC50: half maximal inhibitory concentration, SI: selective index.

Cod	IC50% (ug/ml)	CC50% (ug/ml)	SI: selective index
Extract	24.43 ± 1.02	53.43 ± 0.16	2.1870
NPs	23.03 ± 0.63	61.23 ± 0.48	2.6587

## Conclusion

In this study, *Euphorbia sanctae-catharinae* leaf extract was used for the first time as a green reducing and stabilizing agent for the eco-friendly synthesis of silver chloride nanoparticles (AgCl-NPs). Their formation and characterization were confirmed using UV–Vis, XRD, TEM, and FTIR analyses, showing predominantly spherical particles with sizes ranging from 20 to 50 nm and an average crystallite size of about 24 nm. The AgCl-NPs exhibited strong antibacterial activity against various Gram-positive and Gram-negative bacteria, with MIC and MBC values between 62.5 and 500 µg/mL, and enhanced the efficacy of tetracycline by 21.4–47%. They also showed antiviral activity against HSV-1, inhibiting its replication by 74% at a non-toxic concentration (31.25 µg/mL). These findings highlight their promising potential in biomedical applications to combat infections and antimicrobial resistance.

## Data Availability

The datasets used and analyzed during the current study are available from the corresponding author on reasonable request.
